# Global Regulator AdpA_1075 Regulates Morphological Differentiation and Ansamitocin Production in *Actinosynnema pretiosum* subsp. *auranticum*

**DOI:** 10.3390/bioengineering9110719

**Published:** 2022-11-21

**Authors:** Siyu Guo, Tingting Leng, Xueyuan Sun, Jiawei Zheng, Ruihua Li, Jun Chen, Fengxian Hu, Feng Liu, Qiang Hua

**Affiliations:** 1 State Key Laboratory of Bioreactor Engineering, East China University of Science and Technology, 130 Meilong Road, Shanghai 200237, China; 2 Shanghai Collaborative Innovation Center for Biomanufacturing Technology, 130 Meilong Road, Shanghai 200237, China

**Keywords:** *Actinosynnema pretiosum*, ansamitocin P-3, *ssgA*, AdpA, morphological differentiation

## Abstract

*Actinosynnema pretiosum* is a well-known producer of maytansinoid antibiotic ansamitocin P-3 (AP-3). Growth of *A. pretiosum* in submerged culture was characterized by the formation of complex mycelial particles strongly affecting AP-3 production. However, the genetic determinants involved in mycelial morphology are poorly understood in this genus. Herein a continuum of morphological types of a morphologically stable variant was observed during submerged cultures. Expression analysis revealed that the *ssgA_6663* and *ftsZ_5883* genes are involved in mycelial aggregation and entanglement. Combing morphology observation and morphology engineering, *ssgA_6663* was identified to be responsible for the mycelial intertwining during liquid culture. However, down-regulation of *ssgA*_*6663* transcription was caused by inactivation of *adpA_1075*, gene coding for an AdpA-like protein. Additionally, the overexpression of *adpA_1075* led to an 85% increase in AP-3 production. Electrophoretic mobility shift assays (EMSA) revealed that AdpA_1075 may bind the promoter regions of *asm28* gene in *asm* gene cluster as well as the promoter regions of *ssgA*_*6663*. These results confirm that *adpA_1075* plays a positive role in AP-3 biosynthesis and morphological differentiation.

## 1. Introduction

Ansamitocin P-3 (AP-3) exhibits antitumor activity against various cancer cell lines [1,2,3]. Its derivatives are commonly used as the ‘warhead’ molecule in antibody-conjugated drug (ADC) for the treatment of various solid tumors [4]. AP-3 is a member of various ansamitocin congeners produced by *A. pretiosum*. Ansamitocins are of limited industrial applicability because of their low production yields. In recent decades, considerable efforts have been made to further enhance AP-3 yield to satisfy the industrial demands with medium optimiztion and genetic modifications because of its great pharmaceutical value [5,6,7,8,9,10,11,12]. 

From an industrial point of view, liquid culture is favorable for large scale production of antibiotics. Actinomycetes are usually subjected to submerged fermentation. Unlike other bacteria, *Actinobacteria* remarkably exhibits complex morphology during submerged cultivation. In liquid culture, their mycelium shows filamentous growth, exhibiting dispersed mycelial form or compact mycelial network [13]. It is well studied that different morphological forms lead to various degrees of nutrient and oxygen transfer during the fermentation process [14]. Three types of morphologies-freely dispersed mycelia, open mycelial networks, and compact mycelial network are generally distinguished in submerged cultures [15]. The morphological phenotypes are genetically determined and differ considerably between strains. The formation of unfavorable morphology during liquid-grown cultures may be a major bottleneck hindering the industrial production of antibiotics [16]. Submerged culture studies for different antibiotic production have made efforts to select strains with better growth characteristics. Forming dispersed mycelium helps productivity in the bioreactor for *Streptomyces hygroscopicusin* producing rapamycin [17]. A similar situation was found in previous studies of tylosin and nystatin production in both *Streptomyces fradiae* and *Streptomyces noursei* [18,19]. However, for production of nikkomycin and erythromycin pelleted growth is preferred [20,21]. Additionally, forming dense pellets also contributed to the productivity optimization of *Streptomyces lividans* TK21 for a hybrid antibiotic production as well [22].

Therefore, in order to optimize mycelial morphology in a more targeted and flexible manner, several genetic determinants have been identified that play roles in the control of morphogenesis [23]. The genetic factors include cell-matrix proteins and extracellular polymers. Morphoproteins with specific roles in liquid-culture morphogenesis for apical growth and hypha branching include the cell wall remodeling protein SsgA-like proteins (SALPs) [24], and the cellulose synthase-like protein CslA [25]. Members of the family of SALPs are required to activate cell division in both solid and liquid culture sporulation [26,27,28]. SsgB is the archetypal SALP and functions by colocalizing with SsgA for recruiting FtsZ during aerial hyphae early division stage [29,30,31]. Morphology engineering strategies for *ssgA* gene modification were employed to obtain desirable morphologies and fast growth [32,33].

Interestingly, morphological differentiation caused by *ssgA* transcriptional variations are generally controlled by AdpA [28,34,35]. AdpA is universally present in Actinomycetes, as a global regulator of morphological differentiation and secondary metabolism [36,37]. In *S. griseus*, AdpA positively controls the expression of genes involved in spore formation and aerial mycelium formation, as well as activates the transcription of various genes related to secondary metabolism [38,39]. Whereas, overexpression of the *adpAsx* gene in *S. xiamenensis* 318 had negative effects on cell division genes, such as putative *ssgA*, *ftsZ*, *ftsH*, and *whiB.* Besides, it functions as a bidirectional regulator for the biosynthesis of xiamenmycin and PTMs [40]. To date, AdpA has been proven to contain a C-terminal domain with two helix-turn-helix (HTH) DNA binding motifs [41]. As a global transcription factor, the regulatory relationship between AdpA and target genes has been investigated in *Streptomyces*. AdpA and its orthologs activate or down-regulate genes, including repression of its own transcription, by directly binding to operator regions containing a consensus sequence [38,42,43].

Although *A. pretiosum* is being developed as a sustainable industrial production platform, the genes involved in cell division and morphological development are still poorly investigated. Gene *APASM_4178* was identified as a subtilisin-like serine peptidase encoding gene responsible for mycelial fragmentation [44]. FtsZ protein from *A. pretiosum* as the analogue of β-tubulin, was demonstrated to be the AP-3 binding target. Overexpression of *APASM_5716* gene that encodes FtsZ resulted in AP-3 resistance and overproduction in *A. pretiosum* ATCC 31280 [45]. A two-component signal transduction system, PhoPR homolog was identified in the genome of the *A. pretiosum* X47 strain. PhoP is the response regulator, negatively affecting morphological development and excluding its regulation on the biosynthesis of AP-3 in X47 strain [46]. However, for filamentous microorganisms, the importance of understanding the relationship between mycelial morphological development and antibiotic biosynthesis is nontrivial. In this study, a variant of *A. pretiosum* has been observed to form dense pellets, while the control strain formed loose clumps. In addition, excessive mycelial fragmentation of the control strain was observed early in the fermentation. We investigated several putative genes that may contribute to cell division and pellet architecture. Gene *ssgA_6663* was identified as a key genetic determinant of compact mycelial network formation during solid and liquid cultures. We also characterized the roles of AdpA_1075 in controlling the morphological differentiation and AP-3 production. AdpA_1075 was determined to positively control the biosynthesis of ansamitocin by directly regulating the expression of *asm28*.

## 2. Materials and Methods

### 2.1. Bacterial Strains, Plasmids and Culture Conditions

All plasmids and strains used in this study are listed in Supplementary Table S1. *A. pretiosum* subsp. *auranticum* L40 was derived from *A. pretiosum* subsp. *auranticum* ATCC 31565 by atmospheric and room temperature plasma (ARTP) mutagenesis [12]. Strain L40 and its derivatives in this study were cultivated as described previously [47]. YMG agar plates (for solid culture) and TSBY broth (for liquid culture) were employed for strain culture. For fermentation experiments, strains were cultured in shake flasks at 28 °C for 8 days.

The stability of strain MD15 was tested following the method described by former study [10] with some modifications. In brief, strain MD15 was transferred at 24 h intervals for a total of twenty-five passages in YMG liquid medium. The original strain, fifteenth and twenty-fifth passages were selected to test the fermentation performances stability of strain MD15 in liquid fermentation.

### 2.2. Construction of Recombinant Strains

*CRISPR-Cas9 mediated gene inactivation.* Mutants with gene *ssgA_6663, adpA_1075,* or *asm28* disruption were performed by pCRISPR-Cas9apre with a unified construction process [47]. As an example, the construction of mutant with gene *ssgA_6663* deletion was described briefly. Two homologous arms (upstream 1.2 kb, downstream 1.3 kb) for *ssgA_6663* deletion were amplified and together cloned to *Stu*I-digested plasmid pCRISPR-Cas9apre by NEB DNA Assembly Master Mix (New England Biolabs, Ipswich, MA, USA) to give the pCRISPR-Cas9apreΔ*ssgA*. The ApE software (a plasmid editor, version 2.0.50. https://jorgensen.biology.utah.edu/wayned/ape/, accessed on 5 October 2016) was used to search N20 targeting sequences of sgRNAs. The sgRNA cassettes were cloned into the *Xma*JI/*Sna*BI site of pCRISPR-Cas9apreΔ*ssgA*. The amplification primers used to construct pCRISPR-Cas9apre series gene knockout plasmids are shown in Supplementary Table S2. *E. coli* ET12567 (pUZ8002) was employed to introduce the resulting plasmid into L40. According to protocol described elsewhere [47], the conjugants were induced and screened for the correct constructs by colony PCR and Sanger sequencing (Supplementary Figure S1).

*Construction of plasmids for gene overexpression*. pSETK derived from pSET152 was used to prepare overexpression plasmids of *ssgA_6663*, *adpA_1075*, *ftsZ_5883* or *asm28*. More specifically, the kasOp*-rbs fragment was introduced to *Xba*I/*Eco*RV cloning site of pSET152. Aforementioned genes were amplified from *A. pretiosum* L40 chromosome. The amplicons were cloned into *Nde*I/*Eco*RV site of pSETK, respectively. The obtained recombinant plasmids pSETKssgA, pSETKftsZ, pSETKadpA, pSETKasm28, pSETKftsZ:ssgA, and control plasmid pSETK were individually transferred into *E. coli* ET12567(pUZ8002) and then integrated into the *attB* site of strain MD02 by intergeneric conjugation. The verification of these recombinant strains was performed by PCR (Supplementary Figure S2A–C). To construct the pSETKftsZ:ssgA plasmid, the fragment containing kasOp*-rbs-*ssgA_6663* expression cassette cloned from pSETKssgA plasmid was inserted into pSETKftsZ digested by *Eco*RV to generate pSETKftsZ:ssgA. The verification of gene co-expression was double checked by PCR using two primer pairs 152yz-F/R and ftsZchk-f/ssgAchk-r (Supplementary Figure S2D–F).

### 2.3. RNA Isolation, cDNA Synthesis and Quantitative Real-Time PCR (qRT-PCR)

Total RNA was extracted using a bacterial RNA extraction kit (Jiangsu Cowin Biotech Co., Ltd., Taizhou, China). Isolated RNA was treated by DNase I before being reverse transcribed with cDNA Synthesis Kit (Jiangsu Cowin Biotech Co., Ltd., Taizhou, China). The cDNA templates were amplified in triplicate for each transcription analysis using MagicSYBR Mixture (Jiangsu Cowin Biotech Co., Ltd., Taizhou, China) with primers listed in Supplemental Table S2. The transcription of target genes was determined by RT-PCR using a CFX96 Real-Time System (Bio-Rad, Richmond, CA, USA). *16S rRNA* gene was used for internal normalization. Relative transcript level of genes was quantified by the 2^−ΔΔCt^ method [48].

### 2.4. Determination of AP-3 Production

AP-3 was extracted from the culture supernatant using a previously described method [12]. HPLC analysis of AP-3 was operated on Agilent series 1260 (Agilent Technologies, Inc., Santa Clara, CA, USA) equipped with a SinoChrom ODS-BP C18 column (4.6 mm × 250 mm, 5 μm, Elite, Dalian, China) coupled to UV detector at 254 nm. The column was eluted with 85% methanol and 15% water at 28 °C.

### 2.5. Mycelial Morphology Observation

Mycelial morphology was observed using an optical microscope (Olympus CX 31, Olympus Corporation, Tokyo, Japan). Culture broth (10 μL) was pipetted onto a standard glass slide (25 × 75 mm), dyed with crystal violet. Images were captured under an oil immersion lens (magnification, 100×).

### 2.6. Scanning Electron Microscope (SEM)

Mycelium was harvested by centrifugation, and washed with 0.1 M PBS. The mycelium was resuspended in 2.5% glutaraldehyde solution for 3 h. The fixed samples were then washed twice with 0.1 M PBS. Samples were subjected to gradient dehydration with ethanol solution (50%, 70%, 95% and 100%). Finally, the dehydrated samples analyses were carried out on a S3400-N scanning electron microscopy (Hitachi, Tokyo, Japan).

### 2.7. Heterologous Overexpression of AdpA-1075

Gene *adpA_1075* was amplified with primers 28a1075-F/R. The *adpA_1075* cassette was cloned in *HindI*II/*Nde*I-digested pET28a (+), generating plasmid pET-28a-adpA_1075. The plasmid was transformed into *E. coli* BL21(DE3) for protein overexpression. The generated strain BL21(DE3)/pET-28a-adpA_1075 was cultivated at 37 °C for 2–3 h in 100 mL LB medium containing 50 µg/mL kanamycin until OD600 reached about 0.6–0.8. Isopropyl-β-D-thiogalactoside (IPTG, 0.1 mM) was added after 30 min of cooling at 4 °C and further incubated overnight at 16 °C for AdpA_1075 expression. The cells were harvested and resuspended in 50 mM phosphate buffer solution (pH 7.5). His-tagged AdpA_1075 protein was released from cells by homogenization. Ni SepharoseTM 6 Fast Flow (GE Healthcare Life Sciences, Marlborough, MA, USA) was applied to proteins purification with elute buffer (50 mM phosphate buffer solution, 250 mM imidazole, 500 mM NaCl). The purified His-tagged AdpA-1075 was analyzed by 12.5% sodium dodecyl sulfate-polyacrylamide gel electrophoresis (SDS-PAGE).

### 2.8. Electrophoretic Mobility Shift Assays (EMSA)

EMSA was performed using a Chemiluminescent EMSA Kit (Beyotime Biotechnology, Shanghai, China) according to the manufacturer’s instructions. The two complementary oligonucleotides were annealed, labeled with biotin, and incubated with recombinant proteins in the absence or presence of excess amounts of unlabeled wildtype oligonucleotides. The protein-DNA complexes were separated on 5% polyacrylamide gels and the signals were captured with a Chemiluminescence Imaging System (BG-gdsAUTO 720, Baygene Biotechnol Co., Ltd., Shanghai, China). As a control, for each target gene, excessive unlabeled specific DNA fragments were added to the reactions, resulting in the appearance of free un-shifted probe and demonstrating that the binding was specific.

## 3. Results

### 3.1. Identification of ssgA in A. pretiosum *subsp.* auranticum

**Figure 1:**
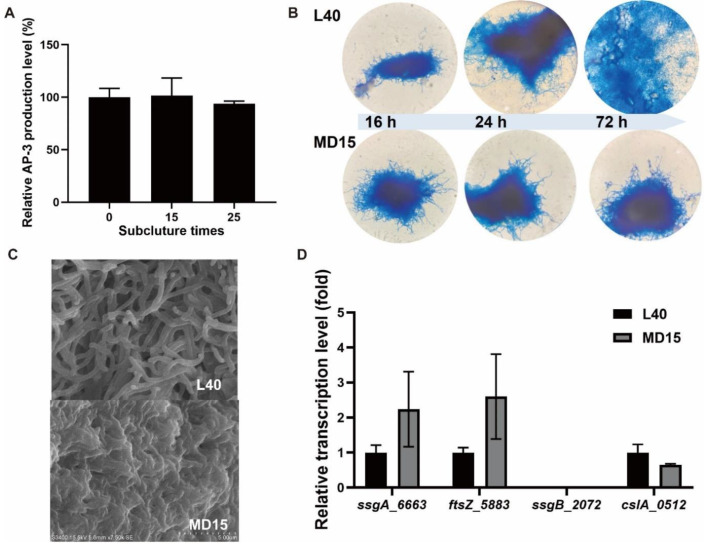
Fermentation stability and morphological variation of mutant MD15. (**A**) Fermentation stability of strain MD15. The strains from original, fifteenth and twenty-fifth subculture were collected to perform liquid fermentation. Fermentation experiments were carried out in three biological replicates. The average AP-3 production of the original MD15 strain was set to 100% as the standard, and the values are means ± SD (standard deviations) of three independent experiments. (**B**) Mycelium morphology changes of strain L40 and mutant MD15 during fermentation. Magnification, 100×. (**C**) Scanning electron micrographs of strain L40 and mutant MD15 on day 3 of YMG solid culture. Scale bar: 5 μm, Magnification, 7500×. (**D**) Transcription levels of the cell division genes of mutant MD15 on the third day of fermentation. The average expression value of genes in the control strain L40 was set to 1 as the standard, and the values are means ± SD of triplicate analyses. Gene expression level was determined by 2^−ΔΔCt^ method.

The previously constructed mutant MD15 with tandem deletion of two gene clusters (cluster T1PKS-15 and T1PKS/NRPS-5) [47] showed excellent fermentation stability (Figure 1A). To better understand the improvement of fermentation performance, the mycelia from culture broth were collected at 16, 24 and 72 h, and aerial hyphae were examined on YMG medium. Scanning electron microscopy (SEM) was used to demonstrate in detail the morphological differences between the mutant strain MD15 and the control strain L40. During the first 24 h of fermentation in both control strain L40 and mutant strain MD15, pellets were formed by aggregative hyphae. Mycelial fragmentation occurred in the control strain L40 at around 72 h, while in contrast, the mycelia of MD15 remained dense pellets with well-developed mycelial boundaries (Figure 1B). Compared with the loosely interwoven mycelial morphological characteristics of L40, the aerial mycelia of MD15 formed a tight mycelial network (Figure 1C). We speculate that gene expression may be altered in strain MD15. SsgA, FtsZ, SsgB, and CslA encoding genes were found, namely *ssgA_6663*, *ftsZ_5883*, *ssgB_2072,* and *cslA_0512*, respectively, in *A. pretiosum* subsp. *auranticum* ATCC 31565 genome. To better understand the role of these genes in mutant MD15 morphological development, the transcription levels of these target genes were measured by qRT-PCR on the third day of fermentation. In mutant MD15, transcription levels of *ssgA_6663* and *ftsZ_5883* were 124% and 160% higher than those in strain L40, respectively (Figure 1D). The transcription of gene *ssgB_2072* was not detected, suggesting that the strain was defective in the initiation of sporulation, as the protein complexes SsgA and SsgB cannot colocalize in aerial hyphae. It has been supported by phenotypic observations. In strain L40 and its derivative, smooth and uncoiled aerial hyphae formed, but no spores (Figure 1C). Negligible changes were observed in *cslA_0512*, which encodes a cellulose synthase-like protein homologue essential for pellet formation [49]. This fact excludes the involvement of *cslA_0512* in the formation of compact pellets in *A. pretiosum*.

Mycelial fragmentation has previously been reported to impede AP-3 production [12,44]. Pelleted growth is preferred for *A. pretiosum* producing AP-3 in submerged cultivation [12]. The sporulation-specific gene *ssgA* directly activates cell division in *Streptomyces* [27,50]. Moreover, the expression of *ssgA_6663* also varies with strain morphology in non-spore producing *A. pretiosum* (Figure 1) [12]. We therefore assume that both *ssgA*_*6663* and *ftsZ_5883* play important roles in controlling the morphogenesis in *A. pretiosum*, especially in the formation of intertwined mycelial network. As reported earlier, filamentous growth and sporulation of actinobacteria require the bacterial tubulin homolog FtsZ [39,51,52]. Overexpression of *ftsZ* gene might also be used to alleviate AP-3 toxicity and improve resistance of *A. pretiosum* to AP-3. Since FtsZ was determined to be the intracellular binding target of AP-3 [45]. A high transcriptional level of *ftsZ_5883* was observed in mutant with a denser mycelial interweaving pattern (Figure 1D). Whether *ssgA*_*6663* and *ftsZ_5883* are involved in specific cellular process and contribute to stable fermentation remains to be investigated.

### 3.2. Deletion of ssgA_6663 Affected the Morphological Differentiation of A. pretiosum

**Figure 2:**
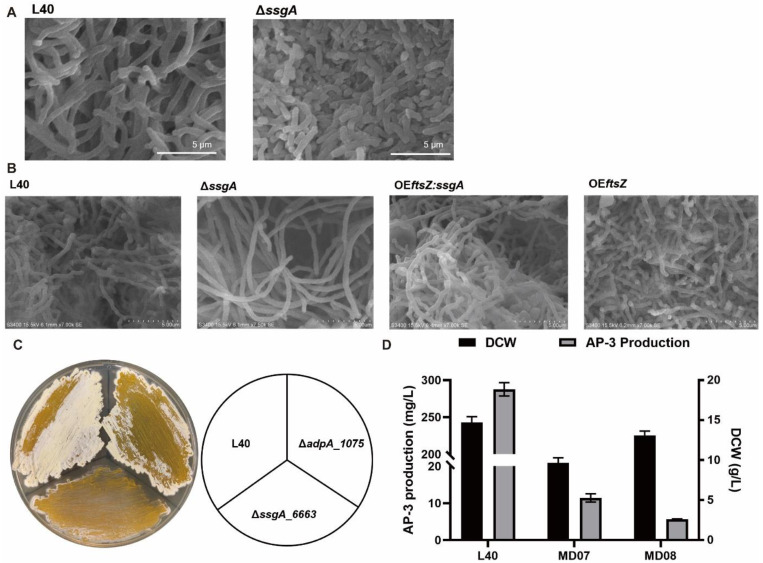
Differences in morphology (**A**–**C**) and AP-3 production (**D**) between mutant strains and L40 strain. (**A**) Scanning electron micrographs of *ssgA_6663* deletion mutant and strain L40 on day 3 of YMG plate culture. Scale bar: 5 μm. Magnification, 7500×. (**B**) Scanning electron microscopic observation of mycelium of strain L40, *ssgA_6663* deletion mutant, strain overexpressing *ssgA_6663* and *ftsZ_5883* in tandem, and mutant with *ftsZ_5883* overexpression in liquid seed culture for 16 h. Scale bar: 5 μm. Magnification, 7500×. (**C**) Phenotypes of control strain L40, *ssgA_6663* deletion mutant, and *adpA_1075* deletion mutant grown on YMG plates at 28 °C (day 3). (**D**) AP-3 production and cell growth of strains L40, MD07, and MD08. MD07, mutant with *ssgA_6663* deletion. MD08, mutant with *adpA_1075* deletion. All fermentation experiments were performed in three biological replicates.

We then investigated the effects of *ssgA*_*6663* deletion on mycelium development and AP-3 production. The length of aerial mycelia of *ssgA*_*6663* disruption mutant was much shorter than that of strain L40 without intertwining (Figure 2A). In submerged cultivation, mycelium of Δ*ssgA*_*6663* mutant developed into dispersed mycelial form (Figure 2B). The short aerial mycelium probably prevented the strain from making biomass from nutrients (Figure 2C). Further HPLC analysis revealed that AP-3 was almost undetected in the fermentation culture of the *ssgA_6663* disruption mutant, and the biomass in the fermentation was also reduced (Figure 2D). However, the introduction of multiple copies of gene *ssgA* may cause an excessive mycelial fragmentation in spore-producing *Streptomyces* [50]. To verify whether *ssgA*_*6663* and *ftsZ_5883* play a positive role in mycelial aggregation and entanglement, these two cell division genes were overexpressed in tandem under the drive of kasOp*. The co-overexpression of *ftsZ_5883* and *ssgA*_*6663* caused the long hyphae to intertwine closely with each other and form clumps, which was more visible than that of the strain overexpressing *ftsZ_5883* alone (Figure 2B). This phenomenon may be consistent with previous reports that secondary metabolism and morphological differentiation can be regulated by global regulator, rather than morphology directly affecting secondary products biosynthesis [35]. The timing of SsgA expression in *Streptomyces* sporulation-specific cell division and morphogenesis can be regulated by global regulator AdpA [50]. As previously reported in *A. pretiosum* ATCC 31280, AdpA-like protein APASM_1021 was found [44]. Gene *adpA_1075* was identified as an AdpA orthologue from three putative AraC family protein encoding genes in *A. pretiosum* subsp. *auranticum* for the high amino acid sequence identity (97.17%) between AdpA_1075 and APASM_1021 (Supplementary Figure S3). We therefore speculate that AdpA_1075 may also play a regulatory role in the L40 strain. Furthermore, the lawns of *ssgA*_*6663-*null mutant on YMG plate exhibited a bald phenotype distinct from L40, whereas deletion of *adpA_1075* had little restriction on the arising of aerial hyphae (Figure 2C). Interestingly, deletion of *adpA_1075* also resulted in a significant decrease in AP-3 production without inhibition of biomass accumulation, suggesting that *adpA_1075* is essential for AP-3 biosynthesis as a pleiotropic transcriptional regulator (Figure 2D).

### 3.3. Overexpression of adpA_1075 Increased the Production of AP-3

**Figure 3:**
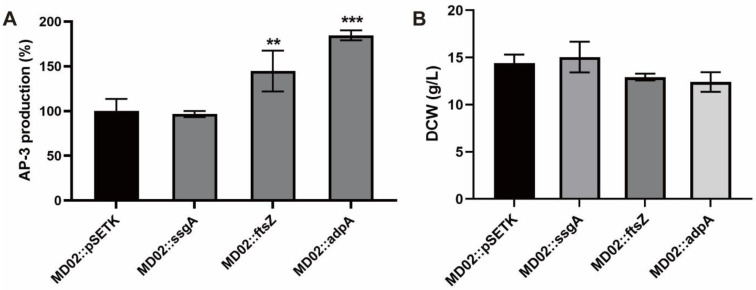
Effects of overexpression of cell division genes on AP-3 production (**A**) and cell growth (**B**). Three biological replicates were performed in fermentation experiments. The values are means ± SD of triplicate analyses. Differences were analyzed by one-way ANOVA, **, *p* < 0.01, ***, *p* < 0.001.

As reported previously, *ssgA* is essential for septum formation in aerial hyphae, a late step in morphological differentiation. Therefore, *ssgA* mutation may not affect the production of secondary metabolites [26,27,28]. In this study, *ssgA*_*6663* or *adpA_1075* was individually overexpressed under the strong promoter kasOp* on the shuttle vector pSETK, to investigate the effects of the enhanced expression of these two proteins on ansamitocin biosynthesis. The fermentation experiments were performed for the recombinant strains overexpressing *ssgA*_*6663*, *ftsZ_5883*, or *adpA_1075*, and the control strain MD02::pSETK (MD02 integrated with the vector pSETK), respectively. The results showed that the overexpression of *ssgA*_*6663* did not improve AP-3 production, which was consistent with what was observed in *S. griseus* [26,28]. As expected, enhanced expression of *ftsZ_5883* improved strain resistance against AP-3 [45], alleviated cell toxicity, and increased AP-3 production by 45% (Figure 3A). Moreover, overexpression of *adpA_1075* increased AP-3 production by 85% without affecting dry cell weight (DCW) at the end of fermentation (Figure 3A,B). Therefore, we may conclude that overexpression of *ssgA*_*6663* only causes morphological changes but does not directly promote AP-3 production. Overexpression of *adpA_1075* may not only regulate ansamitocins biosynthesis, but also regulate strain morphology by controlling the expression of *ssgA_6663*.

### 3.4. AdpA_1075 Is Involved in the Regulation of ssgA_6663 Transcription in A. pretiosum

**Figure 4:**
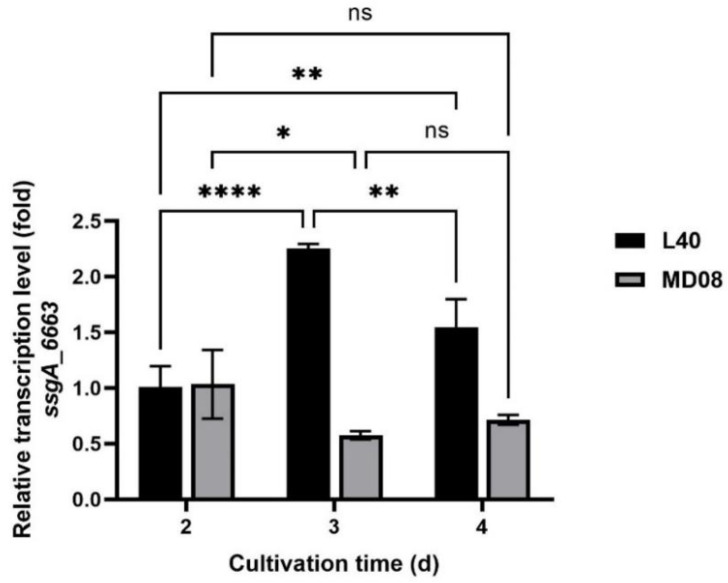
Relative transcription of *ssgA_6663* gene in L40 and MD08 at different culture times. RNA sample data were obtained from days 2, 3, and 4 of fermentation. Gene expression level was determined by 2^−ΔΔCt^ method, and the values are means ± SD of triplicate analyses. Transcription data on day 2 was were set as the baseline for comparison. *16s rRNA* was used as the internal control. Three biological replicates were performed in fermentation experiments. Significant differences were analyzed by one-way ANOVA, *, *p* < 0.05, **, *p*< 0.01, ****, *p* < 0.0001, ns, no significant. MD08, mutant with *adpA_1075* deletion.

The AraC family transcription factor known as AdpA is a global regulator of morphological differentiation and secondary metabolism [42,43,53,54]. Cell division genes are included in members of AdpA regulon, such as *ssgA* which requires AdpA to turn on transcription [28,50,55,56]. Since the fermentation morphology changes caused by *ssgA_6663* inactivation did not directly affect the production of AP-3, we hypothesized that AdpA_1075 might be involved in both *ssgA*_*6663* expression and ansamitocins production. Diametrically opposed transcriptional patterns of *ssgA*_*6663* in *adpA_1075* inactivation mutant seemed to further validate the hypothesis. In parent strain L40, the transcription of *ssgA*_*6663* increased significantly on day 3 of fermentation, but decreased sharply when the strain switched to the stationary growth phase (Figure 4). In contrast, in mutant MD08, *ssgA*_*6663* transcription was down-regulated due to the absence of AdpA_1075 and remained low from the third day of fermentation (Figure 4).

### 3.5. AdpA_1075 Binds to Promoters of ssgA_6663 and asm28

**Figure 5:**
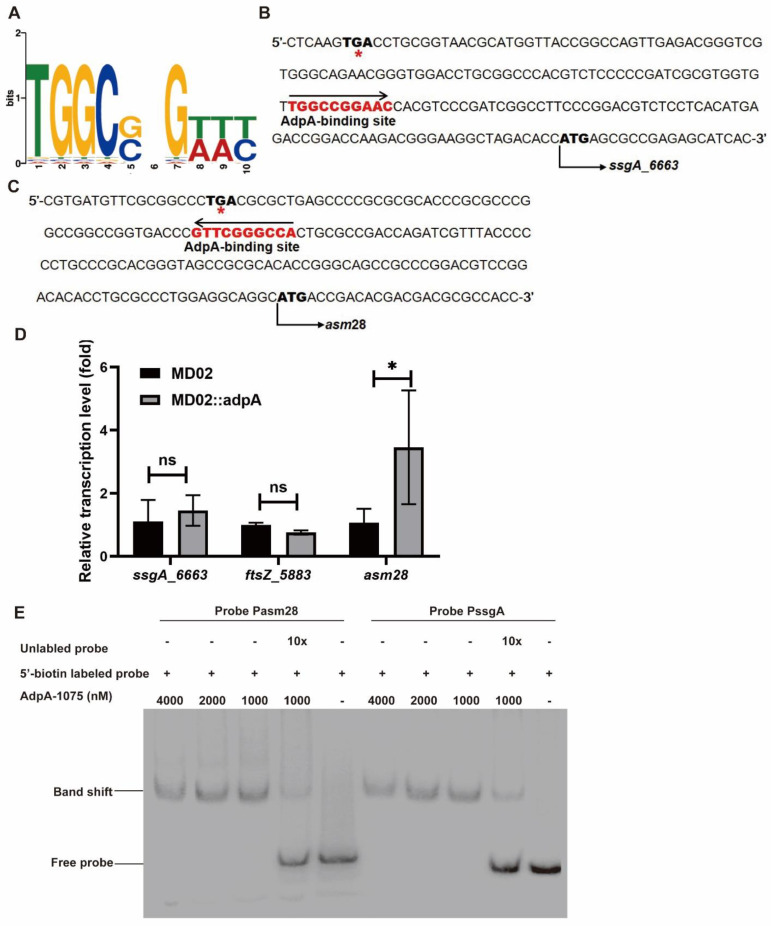
AdpA_1075 binds PssgA and Pasm28. (**A**) The Sequence of AdpA-binding motif. (**B**,**C**) Conserved (shown in red) AdpA-binding motifs in the upstream region of *ssgA*_*6663* and *asm28*. The start codon of *ssgA*_*6663* or *asm28* are shown in bold black. *, stop codons. (**D**) Relative transcription of gene *ssgA_6663*, *ftsZ_5883*, *asm28* in mutant with *adpA_1075* overexpression on day 3 of fermentation. Gene expression level was determined by 2^−ΔΔCt^ method. The average expression value of genes in the strain MD02 was set to 1 as the standard, and the values are means ± SD of triplicate analyses. Differences were analyzed by one-way ANOVA, ns, no significant, *, *p* < 0.05. (**E**) EMSAs of purified AdpA_1075-His_6_ binding to the probes PssgA and Pasm28 labeled with biotin in the upstream region of *ssgA_6663* and *asm28*. 20 ng biotin-labeled probes were incubated for 10 min at room temperature. The experiment was repeated for three times.

The above analyses demonstrated that AdpA_1075 regulated morphological development by controlling the expression of *ssgA*_*6663*. To determine whether AdpA_1075 directly regulated the gene transcription of *ssgA*_*6663*, EMSA was performed. As previously reported, a consensus AdpA-binding sequence such as 5′-TGGCSNGWWY-3′ (S: G or C; W: A or T; Y: T or C; N: any nucleotide) was identified in *S. griseus* (Figure 5A) [57]. Consequently, the upstream region of *ssgA*_*6663* was analyzed by gene sequence alignment and manual correction, according to the reported AdpA-binding motifs in *Streptomyces* and *A. pretiosum* ATCC 31280 [44,57]. A conserved AdpA-binding motif, 5′-TGGCCGGAAC-3′ (in reversed orientation) was identified (Figure 5B). AdpA_1075-His_6_ protein was then purified, and biotin labeled probes were prepared as mentioned above. EMSA results showed that the complex AdpA_1075-PssgA was formed in a protein concentration-dependent manner, confirming that AdpA_1075 bound specifically to the promoter region of *ssgA*_*6663* (Figure 5E). Surprisingly, we not only found an AdpA-binding sequence in the promoter region of *ssgA*_*6663*, but also observed a potential AdpA-binding site 5′-TGGCCCGAAC-3′ (in reversed orientation) in the upstream region of *asm28* (Figure 5C). Compared to the transcriptional changes of *ssgA*_*6663* and *ftsZ_5883* in *adpA_1075* overexpression mutant, transcriptional level of *asm28* in the *adpA_1075* overexpression strain was 3.5 times higher than that in the parent strain (Figure 5D). Unexpectedly, the overexpression of *adpA_1075* did not affect the expression of *ftsZ_5883*. The slight up-regulation of *ssgA*_*6663* indicated that another regulator may be also involved in the regulation of *ssgA*_*6663* transcription. Results of EMSAs showed that Pasm28 probe was shifted when incubated with 1000 nM AdpA_1075-His_6_ (Figure 5E). The above results suggested that AdpA_1075 specifically binds to the promoters of *ssgA*_*6663* and *asm28*, implying that AdpA_1075 controls the transcription of these genes directly. The upstream region of *asm28* is the only potential AdpA binding site on the *asm* gene cluster, as it contains a 10-bp consensus sequence mentioned above. However, the function of *asm28* has not been reported. Therefore, we further investigated the effect of *asm28* gene on AP-3 production. Mutant with *asm28* deletion was constructed by CRISPR-Cas9-mediated vector pCRISPR-Cas9apreΔ*asm28* (Supplementary Figure S4A,B). AP-3 production of the obtained mutant MD19 was only 69% of that of parent strain MD01. However, overexpression of *asm28* promoted ansamitocin biosynthesis and resulted in an approximately 25% increase in AP-3 production, without any effect on DCW of the strain at the end of fermentation (Figure S4D,E). These results demonstrated that AdpA_1075 activated *asm28* and *ssgA*_*6663* on the third to fourth day of fermentation, consistent with the enhanced AP-3 production and mycelial pellet formation in strains.

## 4. Discussion

The mycelial fragmentation of *A. pretiosum* under submerged conditions has long attracted researchers’ attention [12,44,58]. However, in our previous work, pelleted growth was found to be more conducive to the production of ansamitocin [12]. Other studies have revealed that cell-wall remodeling protein SsgA may control the development of fragmentation and promote the growth rate of spore-forming *Streptomyces* strains [32,33]. The absence of SsgB in *A. pretiosum* makes it a non-spore-forming bacterium (Figure 1C,D) [59,60]. Additionally, both mycelial fragmentation and significant biomass enhancement was observed in *ssgA*_*6663*-overexpressing *A. pretiosum* (Figure 2B and Figure 3B). All these results indicate that *ssgA_6663* is not responsible for mycelial fragmentation and AP-3 yield reduction in *A. pretiosum*.

Therefore, we hypothesized that, similar to that reported in *S. limosus* [32], *A. pretiosum* strain may produce large mycelial mat structures in the early growth phase, while the enhanced expression of *ssgA_6663* leads to pellet formation during stationary phase. As expected, a significant increase in the transcriptional profile of *ssgA_6663* was observed in the parent strain L40, followed by a noticeable decrease from day 2 to day 4 of fermentation (Figure 4). However, this transcriptional pattern was disrupted when gene *adpA_1075* was inactivated (Figure 4). 

AdpA as a pleiotropic transcriptional regulator can regulate both morphological differentiation and secondary metabolism [61]. We further investigated the *adpA_1075* gene, encoding an AdpA-like protein. Specific regulation of AdpA-like protein on subtilisin-family serine peptidase encoding gene resulted in a delayed fragmentation, which has been demonstrated in *A. pretiosum* ATCC 31280 [44]. Moreover, the *ssgA_6663* gene has also been identified as being primarily responsible for the formation of mycelial intertwining. Based on these findings, we hypothesized that AdpA_1075, as a global regulator, may comprehensively control the development of multicellular structures and AP-3 biosynthesis during strain fermentation.

Despite the presence of many regulatory genes in the *asm* gene cluster, few studies have been reported on the regulation of ansamitocin biosynthesis. The functions of these regulatory genes were characterized by gene inactivation, complementation, transcriptional analysis, and feeding experiments [62,63,64,65]. For example, negative regulatory gene *asm2* and *asm34*, and positive regulatory gene *asm8* and *asm18* are the putative regulatory genes on the *asm* gene cluster. Whereas, direct evidence for these regulators directly controlled *asm* structural genes was absent. Most of them probably control the expression of genes involved in resistance to xenobiotics, regulation of efflux pumps, and response to stressors [41].

AdpA homologs were well-studied, and have been proven to contain a C-terminal domain with two helix-turn-helix (HTH) DNA binding motifs [37]. In this study, the *adpA_1075* gene was identified as an ortholog of pleiotropic regulator AdpA, and its DNA-binding site is similar to the conserved sequence of the AdpA-binding motifs in many *Streptomyces* species [57]. Earlier work revealed that the expression of APASM_4178, which is responsible for mycelial fragmentation, is specifically regulated by an AdpA-like protein in *A. pretiosum* ATCC 31280 [44]. In this work, the EMSA results also demonstrated the interaction between AdpA_1075 and promoter region of the subtilisin-family serine peptidase encoding gene (data not shown). Our data showed that AdpA_1075 of the *A. pretiosum* L40 strain directly binds to the promoters of the *ssgA_6663* and *asm28*. Therefore, these findings deepen insights into the regulatory roles of the AdpA-like protein in *A. pretiosum*, revealing a high functional similarity to the AdpA homolog in *Streptomyces* reported previously [35].

In this study, an intergenic region containing AdpA-binding motif was identified in the upstream region of *asm28* (Figure 5). Our findings in vitro and in vivo experiments suggested that *asm28* may be the target of AdpA_1075 for regulating ansamitocin biosynthesis (Figure 5 and Figure S4). Moreover, the global regulator *bldA* controls antibiotic production as well, by regulating sporulation and antibiotic production in *Streptomyces* [66]. Generally, the TTA codons are very rare in GC-rich genomes of *Actinobacteria.* TTA-containing genes are usually the cluster-situated regulators of secondary metabolite biosynthesis. In this study, we found that the *asm28* open reading frame (ORF) also contains a TTA codon, indicating that *asm28* might be an important regulatory target during ansamitocin biosynthesis. However, the function of *asm28* gene and its encoded protein remain uncharacterized at present. Follow-up studies are needed to confirm this hypothesis and to elucidate the complex regulatory network in which *asm28* is involved.

## 5. Conclusions

In this study, we have elucidated the function of *ssgA_6663* in mycelial development. *ssgA_6663* can dominate the mycelial intertwining and pellet formation. The silencing of the *ssgB_2072* gene resulted in the absence of SsgB, which may explain the fact that the strain developed into mycelium without sporulation septa.

Additionally, we characterized the regulatory role of AdpA_1075 in *A. pretiosum*. AdpA_1075 acts as a global regulator, affecting morphological differentiation and promoting the biosynthesis of ansamitocin. Our findings provide additional useful evidence for the regulatory mechanism of ansamitocin biosynthesis.

## Data Availability

All data generated or analyzed during this study are included in this article.

## Supplementary Materials

The following supporting information can be downloaded at: https://www.mdpi.com/article/10.3390/bioengineering9110719/s1, Table S1: Strains and plasmids used in this study; Table S2: Primers used in this study; Figure S1: Verification of *ssgA_6663* and *adpA_1075* gene deletion mutant strains; Figure S2: Identification of recombinant strains; Figure S3: Sequence alignment of AdpA-like protein APASM_1021 with AdpA_1075; Figure S4: Effects of *asm28* gene deletion or overexpression.
