# Supplementary material for: Global Regulator AdpA_1075 Regulates Morphological Differentiation and Ansamitocin Production in Actinosynnema pretiosum subsp. auranticum
Source: Bioengineering (Basel). 2022 Nov 21;9(11):719. doi: 10.3390/bioengineering9110719 (PMC9687425; doi:10.3390/bioengineering9110719)
Supplement: Supplementary file 1 [file bioengineering-09-00719-s001.zip › bioengineering-2032800-supplementary.pdf]

Supplementary materials

Global Regulator AdpA_1075 Regulates Morphological Differentiation and Ansamitocin Production in *Actinosynnema pretiosum* subsp. *auranticum*

Siyu Guo [1], Tingting Leng [1], Xueyuan Sun [1], Jiawei Zheng [1], Ruihua Li [1], Jun Chen [1], Fengxian Hu [1], Feng Liu [1,*] and Qiang Hua [1,2,*]

[1] State Key Laboratory of Bioreactor Engineering, East China University of Science and Technology, 130 Meilong Road, Shanghai 200237, China
[2] Shanghai Collaborative Innovation Center for Biomanufacturing Technology, 130 Meilong Road, Shanghai 200237, China
* Correspondence: fengliu@ecust.edu.cn (F.L.); qhua@ecust.edu.cn (Q.H.); Tel./Fax: +86-21-64250972 (Q.H.)

Table S1. Strains and plasmids used in this study.

Strains	Relevant properties	Source or reference
A. pretiosum* subsp. *auranticum		
L40	ARTP mutant derived from ATCC 31565	[1]
MD01	L40 with *asm25* deletion	[2]
MD02	L40 with T1PKS-15 deletion	[2]
MD07	L40 with *ssgA_6663* deletion	This work
MD08	L40 with *adpA_1075* deletion	This work
MD09	L40 with *asm28* deletion	This work
MD15	MD02 with T1PKS/NRPS-5 deletion	[2]
MD19	MD01 with *asm28* deletion	This work
OEssgA	L40 *attB Φ*C31:: pSETKssgA	This work
OEftsZ:ssgA	L40 *attB Φ*C31:: pSETKftsZ:ssgA	This work
MD02::pSETK	MD02 *attB Φ*C31:: pSETK	This work
MD02::ssgA	MD02 *attB Φ*C31:: pSETKssgA	This work
MD02::adpA	MD02 *attB Φ*C31:: pSETKadpA	This work
MD02::ftsZ	MD02 *attB Φ*C31:: pSETKftsZ	This work
MD01:: asm28	MD01 *attB Φ*C31:: pSETKasm28	This work
E. coli		
DH10B	Cloning host	Invitrogen
ET12567/pUZ8002	For intergeneric conjugation	[3]
BL21 (DE3)	Host for expression of protein	Invitrogen
BL21 (DE3)/pET-28a-adpA_1075	Host for expression of AdpA_1075	This work
**Plasmids**		
pCRISPR-Cas9apre	pCRISPR-Cas9 derivative, *rep(pIJ101),* codon-optimized cas9 towards *A. pretiosum*, *Xma*JI-*Sna*BI sgRNA cloning cassette	[2]
pCRISPR-Cas9apreΔ*ssgA*	pCRISPR-Cas9apre derivative, for *ssgA_6663* deletion	This work
pCRISPR-Cas9apreΔ*adpA*	pCRISPR-Cas9apre derivative, for *adpA_1075* deletion	This work

pCRISPR-Cas9apreΔ*asm28*	pCRISPR-Cas9apre derivative, for *asm28* deletion	This work
pSET152	Integrative vector based on *Φ*C31 integrase	[4]
pSETK	pSET152 derivative with *kasOp**	This work
pSETKssgA	pSETK derivative for *kasOp** controlled *ssgA_6663* overexpression	This work
pSETKftsZ	pSETK derivative for *kasOp** controlled *ftsZ_5883* overexpression	This work
pSETKftsZ:ssgA	pSETK derivative for *kasOp** controlled *ftsZ_5883:ssgA_6663* overexpression	This work
pSETKadpA	pSETK derivative for *kasOp** controlled *adpA_1075* overexpression	This work
pSETKasm28	pSETK derivative for *kasOp** controlled *asm28* overexpression	This work
pET-28a(+)	*E. coli* expression vector	Invitrogen
pET-28a-adpA_1075	pET-28a derivative carrying *adpA_1075*	This work

Table S2. Primers used in this study.

Primers	Sequences (from 5′ to 3′) [a, b]
**Primers for the construction and identification of deletion mutants**	
ssgA-UHA-f	CGGGATCTCGTCGAAGGCACTAGAATGAATATTAAATTGTGCTAAGACT T
ssgA-UHA-r	CGGCAGGATTCGAACCTGCGACACCCGCTTTAGGAGAGCGGTGTCAGGT GTCTAGCCTTCCCGTCTTGGT
ssgA-DHA-f	TAAAGCGGGTGTCGCAGGTTCGAATCCTGCCGGGGGCACAAGTAGCCG CTCCCCTCAACCCCCAGGACG
ssgA-DHA-r	GACCCGCGCGGTCGATCCCCGCATATTCCTCACCGTCCCGCTGTGGCTCA
adpA-UHA-f	ATCTCGTCGAAGGCACTAGATTGCGATTCCGTCACGCGCGG
adpA-UHA-r	CGAATCGGGCAACTAGTTTATGGCATGGCCGGATGTTTGCACATG
adpA-DHA-f	CGGCCATGCCATAAACTAGTTGCCCGATTCGGTTTGCTTTCACCCG
adpA-DHA-r	CCCGCGCGGTCGATCCCCGCATATTTCGTTGATCTTGGTGGTGGTGCC
asm28-UHA-f	TGCGGGATCTCGTCGAAGGCACTAGATGCACGGCCTTGCCCCAGC
asm28-UHA-r	GGGCCGACCGGGCTTTCCGGTCGGCACTAGTTTATCATGCCTGCCTCCAG GGCG
asm28-DHA-f	ACACCTGCGCCCTGGAGGCAGGCATGATAAACTAGTGCCGACCGGAAA GCCCGG
asm28-DHA-r	ACCCGCGCGGTCGATCCCCGCATATGTCCTCGTCCATGACGCGGCG
ΔssgAcheck-f	TAGGGCTCGTCGAACAGCGGCTCC
ΔssgAcheck-r	CCATGCTGACCGCGCTGAACTTCAT
ΔadpAcheck-f	TTCACGACCACTTCCGGCATCGCGG
ΔadpAcheck-r	GCTGAGGGTGAGGTTCTCGTCGACGTCGA
Δasm28check-f	ATCGTTTACCCCCCTGCCCGCACG
Δasm28check-r	GCGATCAACAGCCCGTTCTTCGAGCAC
**Primers for amplification of sgRNA cloning cassette**	
ssgA_6663-sg-F	TGGTAGGATCGACGGCCTAGG**GGAGCTGCACTACGAGCCCG** GTTTTAGAGCTAGAAATAGC, *Xma*JI site underlined
adpA_1075-sg1-F	TGGTAGGATCGACGGCCTAGG**GCACGAGCAGGACAACGCGG** GTTTTAGAGCTAGAAATAGC, *Xma*JI site underlined
adpA_1075-sg2-F	TGGTAGGATCGACGGCCTAGG**GCAGCACGAGCAGGACAACG** GTTTTAGAGCTAGAAATAGC, *Xma*JI site underlined
asm28-sg-F	TGGTAGGATCGACGGCCTAGG**GGTGCACCTCGCGTTCGGCG** GTTTTAGAGCTAGAAATAGC, *Xma*JI site underlined

sgRNA-R	TCAGCAGTCCCCGGAACATCGTAGCTGACGCCTACGTAAAAAAAGCACCGACTCGGTGCC, *Sna*BI site underlined
**Primers for the construction and identification of gene overexpression strains**	
ssgA-oe-f	GGAGTTATCTGAGTTGAAGAGGTGACGTCCATATGATGAGCGCCGAGAGCATCACCACGA, *Nde*I site underlined
ssgA-oe-r	GAAACAGCTATGACATGATTACGAATTCGATATCTCAGTTGGTCGAGACCAGCTTGGC, *Eco*RV site underlined
adpA-oe-f	GGAGTTATCTGAGTTGAAGAGGTGACGTCCATATGATGCCACCCCACCGCGTTGTCCTGC, *Nde*I site underlined
adpA-oe-r	GAAACAGCTATGACATGATTACGAATTCGATATCTCACCCGGCGGAGCGGAACGTGGTCCGGTA, *Eco*RV site underlined
ftsZ-oe-f	GGAGTTATCTGAGTTGAAGAGGTGACGTCCATATGATGACGCCCCCGCACAACTACCTCG, *Nde*I site underlined
ftsZ-oe-r	GAAACAGCTATGACATGATTACGAATTCGATATCTCAGCGCCGCATGAACGGCGGCACG, *Eco*RV site underlined
asm28-oe-f	GGAGTTATCTGAGTTGAAGAGGTGACGTCCATATGATGACCGACACGACGACGCGCCAC, *Nde*I site underlined
asm28-oe-r	GAAACAGCTATGACATGATTACGAATTCGATATCTCAGTCGTCGGACCGCGC, *Eco*RV site underlined
152yz-F	GCGTAAGGAGAAAATACCGCATCAG
152yz-R	TTCTGTGGATAACCGTATTACCGCC
ftsZchk-f	CAAGCTCGACATCGGCCGGGAGCT
ssgAchk-r	ATGTGCTCCGCGATGGCGGACAGGA
**Primers for *adpA_1075* heterologous expression amplification**	
28a1075-F	CTGGTGCCGCGCGGCAGCCATATGATGCCACCCCACCGCGTTGTCCTG, *Nde*I site underlined
28a1075-R	TGGTGCTCGAGTGCGGCCGCAAGCTTTCACCCGGCGGAGCGGAACGT, *Hind*III site underlined
**Primers for EMSA probe with biotin labeling**	
Pasm28-f-Biotin	5′Biotin-CGCAGTGGCCCGAACGGGTCCGCAGTGGCCCGAACGGGTCCGCAGTGGCCCGAACGGGTC
Pasm28-r	GACCCGTTCGGGCCACTGCGGACCCGTTCGGGCCACTGCGGACCCGTTCGGGCCACTGCG

PssgA-f-Biotin	5′Biotin-GTGTTGGCCGGAACCACGGTGTTGGCCGGAACCACGGTGTTGGCCGGAACCACG
PssgA-r	CGTGGTTCCGGCCAACACCGTGGTTCCGGCCAACACCGTGGTTCCGGCCAACAC
**Primers used in qRT-PCR analysis**	
ftsZ_5883-RT-F	ATCAAGGTCGTCGGCATCG
ftsZ_5883-RT-R	TCGGCGTCGGACATCAGC
ssgA_6663-RT-F	TCGAGGCGGAGCTGCACTACG
ssgA_6663-RT-R	CTTGGCGAGCTCCCGGTCGAA
adpA_1075-RT-F	CTGCTCAGCGAGGCGGACA
adpA_1075-RT-R	GGGGAACAGCAGGCGGAACT
ssgB_2072-RT-F	TACGCGGTCATCGCCGCGTTC
ssgB_2072-RT-R	AACCACGACGTCGTACGTCCG
cslA_0512-RT-F	GCTCCCCGCAGCGACAAC
cslA_0512-RT-R	AGCCAGGTGCCCCAGGTG
asm28-RT-F	TACGACGGCCTGGAGT
asm28-RT-R	TTGAGCGGCACGAAGT

Note: a, The overlap sequence for DNA assembly is filled with gray background. b, The N20 target sequences of sgRNA are shown in bold.

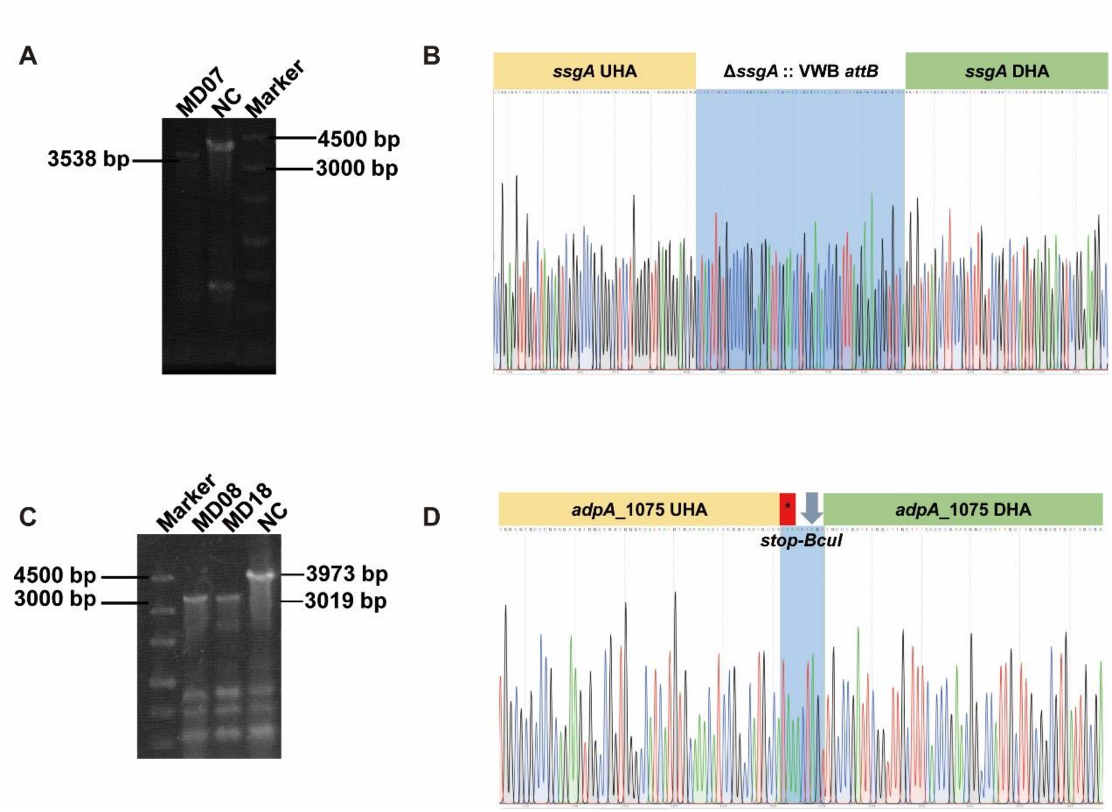

Figure S1. Verification of *ssgA_6663* and *adpA_1075* gene deletion mutant strains. (A) PCR identification of mutant with *ssgA_6663* deletion using test primer pairs ΔssgAcheck- f/r. The PCR product of the mutant MD07 was 3538 bp, NC, negative control. (B) Sanger sequencing chromatograms for the MD07 mutant. Upstream homology arm is shown in yellow, downstream homology arm is shown in green. 411 bp *VWB attB* site sequence replaced *ssgA_6663* gene sequence showing in blue. (C) PCR identification of mutant with *adpA_1075* deletion using test primer pairs ΔadpAcheck-f/r. The PCR products of mutant with *adpA_1075* deletion was 3019 bp, and those of negative control were 3973 bp. NC, negative control. (D) Sanger sequencing chromatograms for mutant with *adpA_1075* deletion. Upstream homology arm is shown in yellow, downstream homology arm is shown in green. Stop codon (TAA) and *BcuI* replaced *adpA_1075* gene sequence showing in blue.

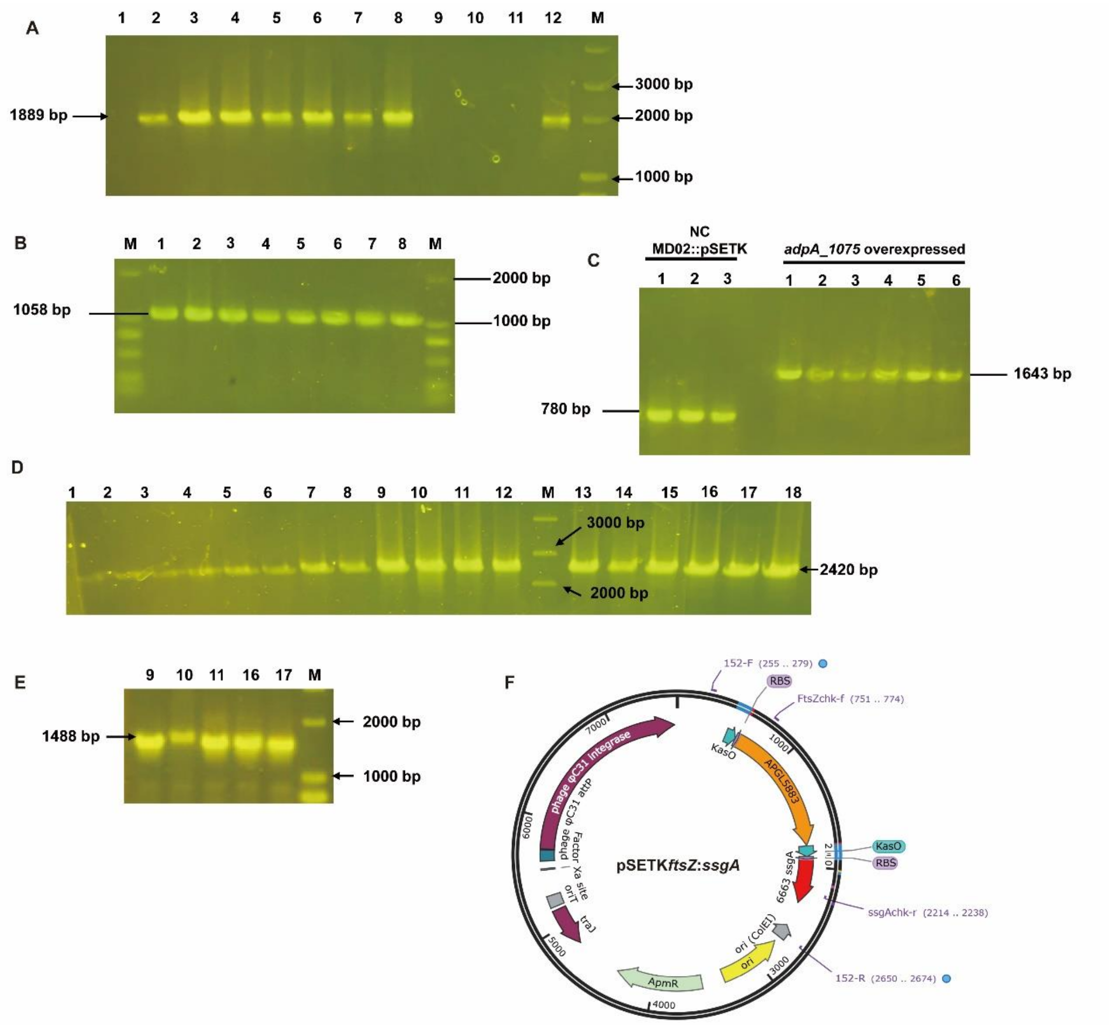

Figure S2. Identification of recombinant strains. (A) PCR identification of mutant with *ftsZ_5883*-overexpressed. (B) PCR identification of mutant with *ssgA_6663*-overexpressed. (C) PCR identification of mutant with *adpA_1075*-overexpressed. Strain MD02::pSETK was selected as negative control. (D, E) PCR identification of strain overexpressing *ssgA_6663* and *ftsZ_5883* in tandem. Primer pairs 152yz-F/R were used in all verification of recombinant strains by PCR. Tandem overexpression of *ssgA_6663* and *ftsZ_5883* was detected by ftsZchk-f/ssgAchk-r for double check. (F) Map of pSETKftsZ:ssgA, the expression of the *ftsZ_5883* and *ssgA_6663* are driven by the *kasOp**.

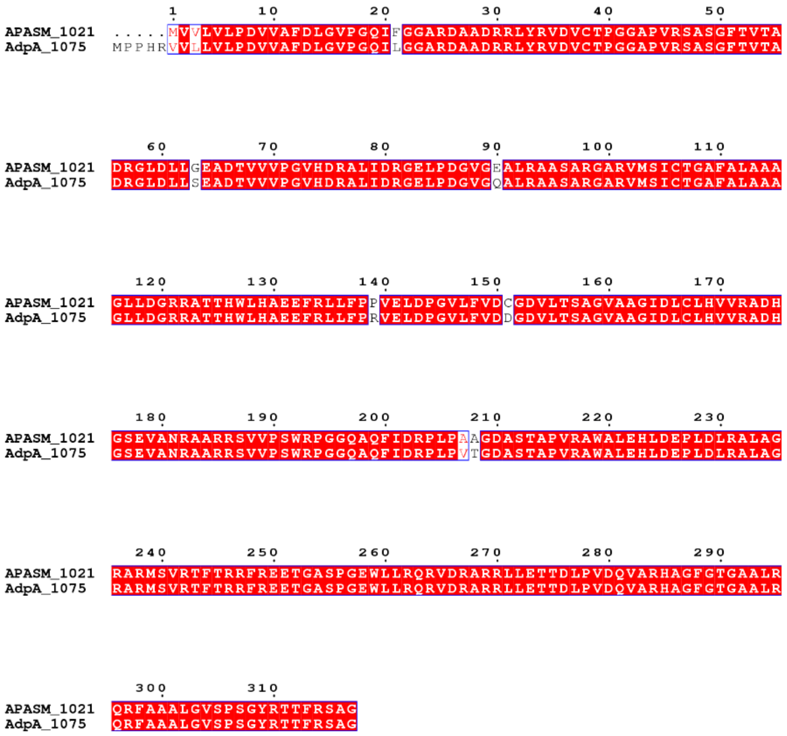

Figure S3. Sequence alignment of AdpA-like protein APASM_1021 with AdpA_1075.

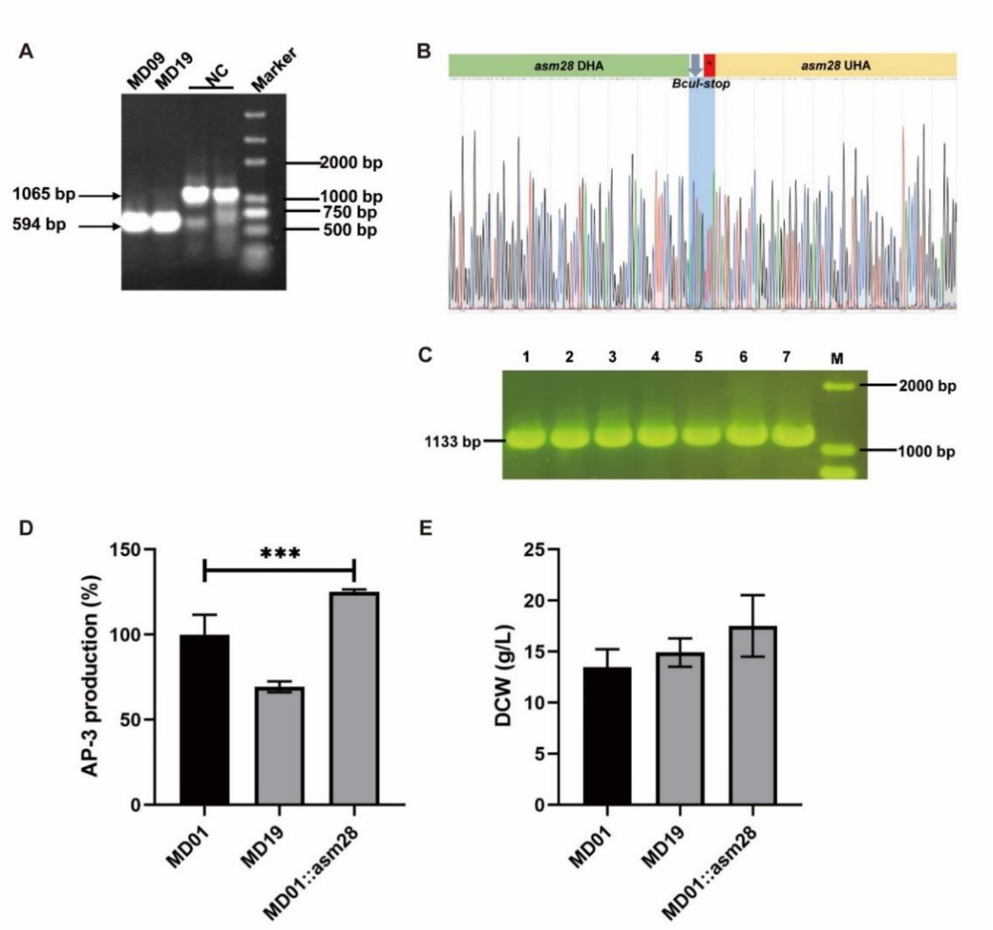

Figure S4. Effects of *asm28* gene deletion or overexpression. (A) PCR identification of mutant with *asm28* deletion strain using test primer pairs Δasm28check-f/r. The PCR products of the mutants were 594 bp, and that of negative control was 1065 bp. NC, negative control. (B) Sanger sequencing chromatograms for mutant with *asm28* deletion. Upstream homology arm is shown in yellow, downstream homology arm is shown in green. A stop codon (TAA) and *BcuI* replaced *asm28* gene sequence shown in blue. (C) PCR identification of mutant with *asm28*-overexpressed, the expression of the *asm28* is driven by the *kasOp**. Primer pairs 152yz-F/R were used in verification of recombinant strains by PCR, the products of the overexpression mutants were 1133 bp. MD19, mutant with *asm28* deletion. MD01 was used as control. Three biological replicates were performed in fermentation experiments. Differences were analyzed by one-way ANOVA, ***, $p < 0.001$.

References

1. Li, J.; Guo, S.; Hua, Q.; Hu, F. Improved AP-3 production through combined ARTP mutagenesis, fermentation optimization, and subsequent genome shuffling. *Biotechnol Lett* **2021**, *43*, 1143–1154, doi:10.1007/s10529-020-03034-5.
2. Guo, S.; Sun, X.; Li, R.; Zhang, T.; Hu, F.; Liu, F.; Hua, Q. Two strategies to improve the supply of PKS extender units for ansamitocin P-3 biosynthesis by CRISPR–Cas9. *Bioresour. Bioprocess.* **2022**, *9*, 90, doi:10.1186/s40643-022-00583-7.
3. Paget, M.S.B.; Chamberlin, L.; Atrih, A.; Foster, S.J.; Buttner, M.J. Evidence that the extracytoplasmic function sigma factor ςE is required for normal cell wall structure in *Streptomyces coelicolor* A3(2). *Journal of Bacteriology* **1999**, *181*, 204–211, doi:10.1128/JB.181.1.204-211.1999.
4. Bierman, M.; Logan, R.; O'Brien, K.; Seno, E.T.; Nagaraja Rao, R.; Schoner, B.E. Plasmid cloning vectors for the conjugal transfer of DNA from *Escherichia coli* to *Streptomyces* spp. *Gene* **1992**, *116*, 43–49, doi:10.1016/0378-1119(92)90627-2.
